# Investigation methylation status of tumor suppressor gene *NR4A1* and *NR4A3* and frequency of rs1569686 polymorphism of *DNMT3B* gene in patients with acute myeloid leukemia

**DOI:** 10.22099/mbrc.2024.51563.2058

**Published:** 2025

**Authors:** Baharan Rahmani, Shahrbano Rostami, Yousef Mortazavi, Mohammad Soleiman Soltanpour

**Affiliations:** 1 Department of Hematology and Blood Banking, School of Allied Medical Sciences, Zanjan University of Medical Sciences, Zanjan, Iran; 2 Cancer Gene Therapy Research Center, Zanjan University of Medical Sciences, Zanjan, Iran.; 3 Hematologic Malignancies Reserch Center, Research Institute for Oncology, Hematology and Cell Therapy, Shariati Hospital, Tehran University of Medical Sciences, Tehran, Iran; 4 Department of Medical Biotechnology, School of Medical Sciences, Zanjan University of Medical Sciences, Zanjan, Iran

**Keywords:** Acute myeloid leukemia, Methylation, Tumor suppressor genes, Polymorphism

## Abstract

Acute myeloid leukemia (AML) is the most frequent type of leukemia among adults. Investigating AML heterogeneity based on DNA methylation can improve clinical diagnosis and prognosis. This study was conducted to investigate *NR4A1* and *NR4A3* gene methylation in fifty newly diagnosed AML patients and fifty healthy controls using Methyl specific PCR (MSP). The frequency of the rs1569686 in the *DNMT3B* was also determined by Tetra primer ARMS PCR. Also, the association between methylation of studied genes and some prognostic marker including mutation of *FLT3* and *NPM* genes, as well as some hematological factors of patients was evaluated. According to the findings, AML patients have a significantly higher prevalence of methylated *NR4A1* and *NR4A3* genes than those without AML. AML patients with un-methylated *NR4A3* had significantly higher frequency of *FLT*-ITD positivity than AML patients with methylated *NR4A3*. Also, there was no significant association between rs1569686 and AML. Finally, the distribution of different genotypes of rs1569686 between AML patients with and without methylation in *NR4A1* and *NR4A3* did not show any significant association. The results found that *NR4A1* and *NR4A3* were hyper-methylated in AML patients. However, rs1569686 polymorphism was not a main contributor to methylation status of studied gene. Future studies should consider other mechanisms influencing the role of *NR4A1* and *NR4A3* hypermethylation in AML.

## INTRODUCTION

Leukemia is the most common malignancy type among blood cancers in different age groups. Leukemia is an unusual phenomenon that is caused by excessive proliferation and incomplete growth of blood cells, which can damage blood cells, bone marrow, and the immune system [1]. Incomplete maturation of myeloid cells is a characteristic feature of AML, a class of bone marrow (BM) and blood cancers. This malignancy is caused by excessive clonal proliferation of a committed stem cell at the level of the colony Forming unit (CFU), which leads to the accumulation of non-functional myeloblasts. Numerous cytogenetic abnormalities that occur in myeloid progenitors are the cause of AML [2, 3].

Population statistics confirm the severity of AML disease [4]. The survival rate for patients with impaired physical conditions over a 5-year period is around 28.3% [3]. The silence of tumor suppressor genes by promoter hypermethylation is a common occurrence in human cancers, making it as a “cancer hallmark” [5]. *NR4A1* and *NR4A3* genes are thought to be tumor suppressors in AML [6]. The *NR4A1* and *NR4A3* gene are located on chromosome 12q13.13 and 9q22, respectively [7, 8]. The loss or downregulation of *NR4A1* and *NR4A3* expression occurs frequently in human AML patients. Recently, it has been reported that there is a downregulation of these two genes, and low expression of *NR4A1* is associated with poor survival in lymphoma patients. The rapid development of AML in mice has been reported to be caused by the deletion of both nuclear receptors NR4A1 and NR4A3. Furthermore, mice with low expression of *NR4A1* and *NR4A3* develop a chronic myeloid malignancy that exhibits pathological features of myelodysplastic/myeloproliferative neoplasms, with rare progression to AML [9]. Methylation can happen at an early stage in the development of tumors and this is detectable in various body fluids, thus, it may be useful in early detection of tumors and prognosis of cancers [10].

The *DNMT3B* is located on chromosome 20q11.2 and encodes DNA methyltransferase 3B (DNMT3B), a protein required for de novo methylation of the genome, establishment of DNA methylation patterns during development, and DNA methylation at centromeric region [11]. The DNA methylation enzymatic activity of DNMT3B may be influenced by polymorphisms in the *DNMT3B*, which could affect the susceptibility to AML [12]. Methylation of promoter and exon regions is widely acknowledged as a significant way to regulate gene expression. It was suggested that genetic variations in *DNMT3B* could regulate the methylation status of other genes associated to AML [13]. In this study, for the first time, the frequency of methylation of tumor suppressor genes *NR4A1* and *NR4A3* were analyzed in patients with AML and control subjects. Moreover, the rs1569686 polymorphism in *DNMT3B* was investigated in study population. 

## MATERIALS AND METHODS

### Participants and sampling:

A group of 50 AML patients (27 males and 23 females) with an average age of 45 years was included in the study. The diagnosis of the patients was verified by employing specific laboratory tests like the use of flow cytometry assay, cytogenetic findings, and peripheral blood analysis. The control group included 50 healthy individuals, comprising 25 men and 25 women who presented themselves for a routine medical evaluation. The subjects were given 5-10 ml of blood for relevant tests. The exclusion criteria for AML patients were individuals with inflammatory, systemic, or underlying disorders, individuals with concurrent malignancies, individuals undergoing specialized therapeutic or immunosuppressive treatment regimens, and individuals who declined participation subsequent to reviewing the informed consent document. Sampling of patients and healthy controls was done with voluntary and informed consent. The study was approved by ethical committee of Zanjan University of Medical Sciences (IR.ZUMS.REC.1402.165).

### DNA extraction and bisulfite treatment:

The DNA extraction process utilized the Favorgen extraction kit (Taiwan, Cat. NO: FABGK001) in accordance with the provided kit instructions. Following the completion of DNA extraction from the samples, the concentration and quality of the nucleic acid were assessed using a nanodrop device (BOECON-1C, Germany) and gel electrophoresis (PNP1000D, Iran), respectively. The Epitec bisulfite kit (Qiagen, USA, Cat. NO: 59104) was employed for bisulfite treatment of DNA samples. As per the manufacturer's instructions, the columns included in the kit exhibited a binding capacity of 1000-2000 ng of bisulfite-modified DNA. The bisulfite DNA samples that were acquired were stored at temperatures ranging from -15 to -30°C until the commencement of the tests.

### Investigation of NR4A1 and NR4A3 methylation:

MSP primers were designed and ordered from metabion (metabion international AG, Germany) (Table S1). For *NR4A1* methylation assays, CpG island 87 in promoter region and for *NR4A3 *methylation assays CpG island 400 in exon 3 was used for primer designing by “MethPrimer” online software (https:// www.urogene.org/methprimer/). The specificity of designed primer were checked using online “BiSearch Primer Design and Search Tool” (http://bisearch.enzim.hu/). The sequence and annealing sites of appropriate primers are presented in Figure S1. The reaction mixture consisted of 10 μl of 2X Hot start Master mix (Ampliqon, Denmark), 1 μl (0.50 µM) of each primer, 2 μl (~100ng) of bisulfite converted DNA, and 6 μl of sterile distilled water. The temperature profile encompassed an initial denaturation stage at 95°C for a duration of 10 minutes, followed by 35 cycles consisting of denaturation at 95°C for 30 seconds, annealing at the temperature (59°C/30 seconds for *NR4A1* Un-methylated, 61°C/30 seconds for *NR4A1* Methylated, 60°C/30 seconds for *NR4A3* Un- methylated, 64°C/30 seconds for *NR4A3* Methylated) and extension at 72°C for 30 seconds. Furthermore, the ultimate amplification process was conducted at a temperature of 72°C for 7 minutes. Appropriate methylated and unmethylated DNA control set (EpiTect Control DNA and Control DNA Set, Qiagen, Germany) were included in each PCR run to ensure of accuracy of results. Following the completion of the reaction, electrophoresis was conducted using a 3% agarose gel.

### Investigation of rs1579686 (-579 G>T) of the DNMT3B with Tetra ARMS-PCR:

After designing the primers, their synthesis was entrusted to Metabion Company (Table S1). The sizes of the products were found to be 337 bp for the entire outers, 217 bp for the T (varaint allele), and 165 bp for the G (ancestral allele). The final volume of PCR for investigating genetic polymorphism was considered to be 20 μl and includes 10 μl of hot start Mastermix 2X (Ampliqon, Denmark), 1.25 μl (0.625 µM) of FI primer, 0.25 μl (0.125 µM) of RI primer, 0.5 μl (0.250 µM) of each outer primer (forward & reverse), 1μl (=100 ng) of DNA and 6 μl of PCR grade water. The temperature profile consisted of 1 initial denaturation step at 95°C for 10 minutes, 35 cycles of 30 seconds denaturation at 95°C, 30 seconds annealing at 63°C, and 30 seconds extension at 72°C. Also, the final amplification was done at 72°C for 7 minutes. After the reaction, electrophoresis was performed on 2.5% agarose gel (Fig. S2).

### Data analysis:

The data were assessed using either the Chi-square test or Fisher's exact test. It is important to acknowledge that statistical significance was determined by considering p-values that were less than 0.05. SPSS for windows version 16.0 (released 2007,Chicago. SPSS Inc.) was employed as the statistical analysis software.

## RESULTS

The demographic, clinical and laboratory characteristics of the study population are presented in Table S2. Statistical analysis indicated no significant difference in the sex distribution and median age between the two groups. The methylation frequency of both genes was significantly higher in patient than control group (Table 1). Regarding the *NR4A1*, the methylation frequency was 52.0% and 30.0% in patients and controls, respectively. While, the frequency of *NR4A3* gene methylation was 68.0% and 42.0% in patient and control groups, respectively. 

Some of the demographic and laboratory parameters of AML patients was evaluated based on methylation status of *NR4A1* and *NR4A3* genes. Results indicated no significant association between age, sex, median platelet count, mean hemoglobin concentration and AML FAB classification and the methylation status of the *NR4A1* and *NR4A3*. However, the median WBC count was significantly higher in AML patients with methylated *NR4A1* than those with unmethylated *NR4A1*. Conversely, AML patients with unmethylated *NR4A3* had significantly higher median WBC count than those with methylated *NR4A3* (Table 2).

**Table 1:** Frequency of methylation in *NR4A1* and *NR4A3* genes in patient and control groups

**Methylation status**	**Patients ** **n=50 (%)**	**Controls ** **n=50 (%)**	**OR**	**95% CI**	***p***
Un-methylated NR4A1	24 (48.0)	35 (70.0)	1.0	-	-
Methylated NR4A1	26 (52.0)	15 (30.0)	2.58	1.09-5.82	0.041
Un-methylated NR4A3	16 (32.0)	29 (58.0)	1.0	-	-
Methylated NR4A3	34 (68.0)	21 (42.0)	2.94	0.015	0.015

**Table 2:** Demographic, clinical and laboratory condition of AML patients based on methylation status of *NR4A1* and *NR4A3* genes

**Patient parameters**	**Status of ** ***NR4A1*** **Methylation**		***p***	**Status of ** ***NR4A3*** **Methylation**		***p***
	**M (26)**	**U (24)**		**M (34)**	**U (16)**	
Male/Female	13/13	14/10	0.554	19/15	8/8	0.93
Median WBC, µL^-1^ (range)	15040 (1130-120000)	6600 (1140-157200)	0.042	6600 (1140-120000)	18240 (1130-157200)	0.031
Median hemoglobin, g/dL (range)	8.4 (4.9-14.5)	8.20 (5.5-12.0)	0.672	8.10 (4.9-14.5)	8.70 (5.5-11.9)	0.867
Median platelets, µL^-1^ (range)	41000 (700-380000)	48000 (1200-290000)	0.771	41000 (7000-350000)	82000 (11000-380000)	0.137
Age <60/ ≥60 years	14/12	14/10	0.782	21/13	7/9	0.563

The frequency of TT genotype and GT heterozygous genotype in patient group was 22% and 36%, respectively. While, in the control group these values were 12% and 36%, respectively. As shown in Table 3, no significant differences were observed regarding the genotype’s distribution of rs1569686 between the two groups. Also, the T allele frequency in patient and control group (40% vs. 30%) did not show any significant differences. 

**Table 3:** Assosictaion between *DNMT3B* rs1569686 (-579G>T) polymorphism and AML

**Genotypes/Alleles**	**Patients ** **n=50 (%)**	**Controls ** **n= 50 (%)**	**OR**	**95% CI**	***p***
GG genotype	21 (42.0)	26 (52.0)	1.0	-	-
GT genotype	18 (36.0)	18 (36.0)	1.23	0.51-3.06	0.66
TT genotype	11 (22.0)	06 (12.0)	2.27	0.72-7.69	0.25
G allele	60 (60.0)	70 (70.0)	1.0	-	-
T allele	40 (40.0)	30 (30.0)	1.56	0.88-2.82	0.18

We investigated the impact of different genotypes of *DNMT3B* rs1569686 polymorphism on methylation status of studied genes in whole study population. As indicated in Table 4, the GT and TT genotype distribution of rs1569686 polymorphism did not significantly differed between methylated and un-methylated state of *NR4A1*. Similar results were also obtained for *NR4A3* gene. The frequency of *NPM* and *FLT3*-ITD mutations in our AML patients were 38% and 34%, respectively. 

As shown in Table 5, the prevalence of *FLT3-ITD* positivity were not significantly different between patients with methylated and un-methylated *NR4A1*. Conversely, *FLT3-*ITD positivity were more common among patients with un-methylated *NR4A3* than those with methylated *NR4A3*. The positivity of *NPM* mutation between patients with methylated and un-methylated *NR4A1* gene were not statistically significant. Similarly, the positivity of *NPM* mutation did not differed significantly between patients with methylated and un-methylated *NR4A3* gene. 

**Table 4:** The association between different genotypes of *DNMT3B* rs1569686 (-579G>T) polymorphism and methylation status of *NR4A1* and *NR4A3* genes in study participants

**Genotypes**	**NR4A1 methylated ** **n=41 (%)**	**NR4A1** **Un-methylated ** **n=59 (%)**	**NR4A3** **Methylated ** **n=55 (%)**	**NR4A3** **un methylated ** **n=45 (%)**
GG genotype	17 (41.46)	30 (50.85)	30 (54.55)	17 (37.78)
GT genotype	15 (36.58)	21 (35.60)	17 (30.91)	19 (42.22)
TT genotype	09 (21.96)	08 (13.55)	08 (14.54)	09 (20.00)
Statistical analysis	$x^{2}$ =1.46, df=2, *p*=0.48		$x^{2}$ =2.79, df=2, *p*=0.24	

**Table 5:** The association between *FLT3* and *NPM* mutation status and methylation frequency of *NR4A1* and *NR4A3* genes in patient group

***FLT3*** ** and ** ***NPM*** **Mutation status**	***NR4A1 *** **methylated** **n=26 (%)**	***NR4A1*** **Un-methylated** **n=24 (%)**	***NR4A3*** ** methylated** **n=34 (%)**	***NR4A3*** **Un-methylated** **n=16 (%)**
*FLT3*-ITD Positive (n= 17)	06 (23.08)	11 (45.83)	07 (20.59)	10 (62.50)
*FLT3*-ITD Negative (n=33)	20 (76.92)	13 (54.17)	27 (79.41)	06 (37.50)
Statistical significance	OR=2.82, 95% CI (0.79-8.84), *p*=0.139		OR=6.43, 95% CI (1.84-21.69), *p*=0.009	
*NPM* Positive (n= 19)	09 (34.62)	10 (41.67)	12 (35.29)	07 (43.75)
*NPM* Negative (n=31)	17 (65.38)	14 (58.33)	22 (64.71)	09 (56.25)
Statistical significance	OR=1.35, 95% CI (0.40-3.89), *p*=0.771		OR=1.43, 95% CI (0.42-4.50), *p*=0.755	

## DISCUSSION

The main findings of the present study were that the frequency of methylated *NR4A1* and *NR4A3* genes were more common in AML patients than control group. Also, the *DNMT3B* rs1569686 has no effect on AML development and on methylation status of *NR4A1* and *NR4A3* genes. Moreover, *FLT3*-ITD mutation was more common among patients with un-methylated than methylated *NR4A3*. Methylation of the *NR4A1* has been investigated in various cancers using different methods. In our study, the methylation rate of the *NR4A1* was reported 52.0% in patients. In Safe et al.'s study, in which the *NR4A1* methylation was investigated using the MAT technique, a relatively higher methylation rate (Mat score: 5.14054) was reported [16, 17]. 

In our research, the methylation rate of the *NR4A3* was estimated to be 68.0% in AML cases and 42% in control subjects. A study examined the methylation level of the *NR4A3* gene by the bisulfite sequencing technique. They reported the methylation levels of *NR4A3* in AML cell lines to be above 80%, whereas the control group exhibited methylation levels ranging between 40% and 50% [15]. Our current results are in accordance to some extent with the results of this study [15].

A recent study, indicated that *NR4A3* silencing is thought to impair the differentiation capacity of myeloid cells while the *NR4A1* abrogation is thought to confer a survival and proliferation advantage to myeloid cells. This study indicated the cooperation of these two tumor suppressor genes in AML induction and provided a possible mechanism for classical two-hit theory in leukemogenesis [14]. In agreement with Lin et al study, the present study identified higher methylation rate of *NR4A1* and *NR4A3* in AML patients and supported the two-hit theory in AML leukemogenesis [20]. Reduced expression and hyper-methylation of *NR4A1* and *NR4A3* are commonly encountered phenomenon in blood cancers. However, in a study, no hypermethylation was reported in the *NR4A1* and *NR4A3* genes in aggressive lymphomas [18]. Many factors including differences in disease nature, variations in study groups, differences in survey techniques and the environmental factors may influence the reported methylation frequency in various cancers [19]. 

Our present finding on *DNMT3B* rs1569686 polymorphism with susceptibility to AML is in accordance with one study [23] and is in discordance with some [12, 13] studies. Ethnic difference of studied population, sample size variability, population-specific linkage disequilibrium between markers and causal variants and interactions between genes and environmental factors may explain these inconsistent results [24]. It should be noted that our present result is not similar with a recent study investigated the association bwteen the rs1569686 and risk of bladder cancer [25]. DNA methylation may be affected by *DNMT3B* and its genetic polymorphisms. Moreover, our results indicated no significant impact of rs1569686 polymorphism on methylation status of the *NR4A1* and *NR4A3* genes in AML disease.

The frequency of *FLT3*-ITD and *NPM* mutation in present study were similar to reported frequency of these mutations in previous study [21]. No association was seen between *NPM* mutation and methylation status of *NR4A1* or *NR4A3*. However, *FLT*-ITD mutation was more common among un-methylated than methylated *NR4A3* gene in AML. *FLT3*-ITD has identified as a poor prognostic factor in AML. It seems that methylated *NR4A3* is only involved in the development of AML but lacks a significant impact on severity and prognosis of disease. It is reported that there is no correlation between *FLT3* mutation and DNA methylation profile in AML patients [22]. On the contrary to our study, they discovered an association between *NPM1* mutation and both hypo- and hyper-methylation in AML patients.

In the present study, no significant association was seen between age, sex, median platelet count, mean hemoglobin concentration and AML FAB classification with the methylation status of the *NR4A1* and *NR4A3*. However, the median WBC count was significantly higher in AML patients with methylated than those with un-methylated *NR4A1*. Such an association imply the importance of methylated *NR4A1* in determining disease severity [26]. The present study faced several limitations, including the lack of access to the patient flow cytometry results, which could have provided more detailed insights into the cellular characteristics of the samples. Additionally, the limited sample size may affect the generalizability of the findings. The inaccessibility to various subtypes of AML potentially limited the comprehensiveness of the results. Data collection from a single center may not represent the broader population. 
