# Supplementary material for: Investigation methylation status of tumor suppressor gene NR4A1 and NR4A3 and frequency of rs1569686 polymorphism of DNMT3B gene in patients with acute myeloid leukemia
Source: Mol Biol Res Commun. 2025;14(2):149–56. doi: 10.22099/mbrc.2024.51563.2058 (PMC11865936; doi:10.22099/mbrc.2024.51563.2058)
Supplement: Supplementary file 1 [file MBRC-14-149-s001.pdf]

Table S1. Primer information of methylated and un-methylated NR4A1 and NR4A3 genes and rs1569686

**Genes**	Primers	Sequences	Tm (°C)	Product length (bp)
**Methylated NR4A1**	Forward	GGTTTGTTTTGATCGTTTAGTAGC	60	
	Reverse	CACTCTCCAATTACTCCCGAA	59	142
**Unmethylated NR4A1**	Forward	GTTTGTTTTGATTGTTTAGTAGTGG	59	
	Reverse	CTCACTCTCCAATTACTCCCAAA	61	143
**NR4A3 Methylated**	Forward	GTTGGATTCGTCGATGAAGGCG	64	
	Reverse	CGTCGAATCGTAACCGAAATAATAACCG	67	161
**NR4A3 Unmethylated**	Forward	TGTTGGATTTGTTGATGAAGGTGG	62	
	Reverse	ACCATCAAATCATAACCAAAATAATAACCA	63	165
**Rs 1569686 Outer**	Forward	GTGGTGTGAGTGACCTGGAG	63	
	Reverse	GCAACATTATGGGCACACAG	58	327
**Rs1569686 Inner**	Forward	CTTGGAAAACTCGGTTTCAACT	58	
	Reverse	AAAACTTCAGGGCATAAATCTCC	59	217, 165

Table S2. The demographic, clinical and laboratory characteristics of the study population

**Hematologic factors**		**Hb** (g/dl)		**WBC count** ($\times 10^3/\mu L$)	**PLT count** ($\times 10^3/\mu L$)
AML patients	Median	8.3		12450	46500
	minimum	4.9		113	7000
	Maximum	14.5		157200	380000
Control	Median	13.2		5650	246000
	minimum	9.8		3900	148000
	Maximum	16.5		8800	435000
**FAB classification**		**AML-M1**	**AML-M2**	**AML-M4**	**AML-M5**
The frequency in patients		n=23<br>46%	n=9<br>18%	n=10<br>20%	n=8<br>16%
**Sex**		**Male**		**Female**	
The frequency in patients		n=27<br>54%		n=23<br>46%	
The frequency in control		n=25<br>50%		n=25<br>50%	
P-value=0.84					
**Median age**		**≥60**		**<60**	
The frequency in patients		n=22<br>44%		n=28<br>56%	
The frequency in control		n=17<br>34%		n=33<br>66%	
p-value=0.67					

NR4A1 Gene

(upstream)[52050755-52051096], CPG island 87 was selected.

Bisulfite conversion M:

5’TTTTGTTGGGGAAGTCGCGTTTTGTGTATTAGTTGCGTTTAGGGTTGAGGTGAGGGCGTAGGTTTTTTAGGGTGTGTTCGAATT
GTTCGTTTTAGTTCGCGGTTTGTTTTGATCGTTTAGTAGCGGTAGTAGCGATATTTTAGGGTTTTAGGAAGGGTTTGGGAAGGTG
TAAAGGCGGGGTTAGGTTCGGAGGGAGTCGGAGGGATCGGGCGCGGTTGGTTTTCGGGAGTAATTGGAGAGTGAGGAGATTT
TTATTCGGGGAAGTTTCGCGGTCGCGTTTTTATAGCGTTTTTTTTTCGGGTTTTGGTTTTTTCGTTGGTTATTTTGGATTTGGGGGTT
TTTA 3’

Bisulfite conversion U:

5’TTTTGTTGGGGAAGTTGTGTTTTGTGTATTAGTTGTGTTTAGGGTTGAGGTGAGGGTGTAGGTTTTTTAGGGTGTGTTTGAATT
GTTTGTTTTAGTTTGTGGTTTGTTTTGATTGTTTAGTAGTGGTAGTAGTGATATTTTAGGGTTTTAGGAAGGGTTTGGGAAGGTGT
AAAGGTGGGGTTAGGTTTGGAGGGAGTTGGAGGGATTGGGTGTGGTTGGTTTTTGGGAGTAATTGGAGAGTGAGGAGATTTT
TATTTGGGGAAGTTTTGTGGTTGTGTTTTTATAGTGTTTTTTTTTGGGTTTTGGTTTTTTTTGTTGGTTATTTTGGATTTGGGGGTTT
TTA3’

NR4A3 gene

(exon3) [99819867-99820227], CPG island 400 was selected.

Bisulfite conversion M:

5’TCGAATCGAGTTTAAGATGGCGATTTCGTTTTCGTTTTTTTCGTATTTTCGGCGGGGTTAGGGCGTTTTTTTACGCGGCGGGTTC
GCGTTCGAGATTCGGTTAAAAATAGTTAGGTTCGATTATATTTGGTTCGGTCGGATTTTGGCGCGGTCGCGCGTTTTTCGTTTCGT
TTCGTTTCGTTTCGCGCGTTTTGTTTCGTTTCGCGCGGCGTTGAATGGAGAAGGGGCGGGGGTGATCGAGGGGAATTTATTTCG
TTATTTGCGGCGCGCGTCGGCGGCGGGTTTCGGTTAATCGTCGAATTTAGTAATATCGTTTGCGTTAATTACGCGTTTCGCGTGCG
TTAGCGTCGCGCGGT3’

(exon3) [99828457-99828792]

Bisulfite conversion U:

5’TTATTGTTTATTTTTATTATGTTGGTTTTTTTTTTTGTAGGTGGGGGTGTTATGGGATGAGGTATTGTTTTTGGTGTTTGGTTGTATT
GTATTTGGTTTGTTGTTGGATTTGTTGATGAAGGTGGTTTTTATGGTGGTTGGTGTGTGTTTTTTGTTTTTTATTTAAGTTTTTGT
TGTTGTATTTTTTTGTGTTTAGTTTGGTTGGTGGTTATTATTTTGGTTATGATTTGATGGTTGTTGTTGTGTTTAGTTTGTTGTTGGG
AGTTGTAGTTGTTGTGGGTAGTTAGGTTGTTGTGTTTGAGAGTTATTTGTATGGGTTGTTGTTGGTTAAGAGG3’

Figure S1. The sequence and annealing sites of Methylated (M) and Unmethylated (U) primers of *NR4A1* and *NR4A3* genes

(The highlighted areas are the place that primers specifically bind.The red Ts are cytosines that have been converted to thymine during the bisulfite process.)

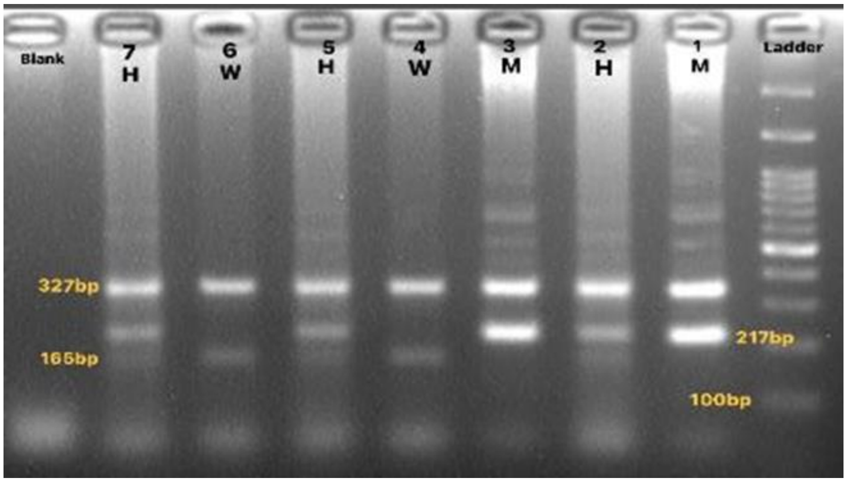

Figure S2. Gel electrophoresis of rs1569686 polymorphism of DNMT3B gene that includes negative control(blank), ladder and patient's sample. "M" is mutant homozygous; "H" is heterozygous and "W" is normal homozygous
